# Thirty-Three Years Follow-Up of a Greek Family with Abetalipoproteinemia: Absence of Liver Damage on Long-Term Medium Chain Triglycerides Supplementation

**DOI:** 10.3390/jpm15080354

**Published:** 2025-08-04

**Authors:** John K. Triantafillidis, Areti Manioti, Theodoros Pittaras, Theodoros Kozonis, Emmanouil Kritsotakis, Georgios Malgarinos, Konstantinos Pantos, Konstantinos Sfakianoudis, Manousos M. Konstadoulakis, Apostolos E. Papalois

**Affiliations:** 1 GI Unit, Metropolitan General Hospital, Holargos, Greece, 264 Mesogeion Ave., Holargos, 155 62 Athens, Greece; aretiman@gmail.com (A.M.); gmalgarinos@yahoo.co.uk (G.M.); 2 Hellenic Society of Gastrointestinal Oncology, 354, Iera Odos str., Haidari, 124 61 Athens, Greece; papaloisapostolos@gmail.com; 3 Hematology Laboratory-Blood Bank, Aretaieion Hospital, School of Medicine, National and Kapodistrian University of Athens, 76 Vas. Sophias Av., 115 28 Athens, Greece; teopittaras@yahoo.gr; 4 2nd Department of Surgery, School of Medicine. National and Kapodistrian University of Athens, 76 Vas. Sophias Av., 115 28 Athens, Greece; kozonis24@yahoo.com (T.K.); ekritsotakis2020@gmail.com (E.K.); mkonstad@med.uoa.gr (M.M.K.); 5 Centre for Human Reproduction, Genesis Athens Clinic, 14-16, Papanikoli, 152 32 Athens, Greece; info@pantos.gr (K.P.); sfakianosc@yahoo.gr (K.S.)

**Keywords:** abetalipoproteinemia, malabsorption syndrome, lipids, hereditary syndromes, nutrition, MCT oil

## Abstract

**Background:** The long-term clinical and laboratory results of a 33-year follow-up of a Greek family with abetalipoproteinemia (ABL) are described. Case Report: The patients (two brothers and their sister, aged 57, 49, and 62 years, respectively) are still alive, being under close surveillance. In two of the three patients, diarrhea appeared in early infancy, while in the third, it appeared during adolescence. CNS symptomatology worsened after the second decade of life. At the same time, night blindness appeared in the advanced stages of the disease, resulting in almost complete loss of vision in one of the male patients and severe impairment in the other. The diagnosis was based on the clinical picture, ophthalmological findings, serum lipid estimations, and presence of peripheral acanthocytosis. All patients exhibited typical serum lipidemic profile, ophthalmological findings, and acanthocytes in the peripheral blood. During the follow-up period, strict dietary modifications were applied, including the substitution of fat with medium-chain triglycerides (MCT oil). After 33 years since the initial diagnosis, all patients are alive without any sign of liver dysfunction despite continuous use of MCT oil. However, symptoms from the central nervous system and vision impairment worsened. Conclusion: The course of these patients suggests that the application of a modified diet, including MCT oil, along with close surveillance, could prolong the survival of patients without significant side effects from the liver.

## 1. Background

Abetalipoproteinemia (ABL; OMIM 200100) is an extremely rare recessive character genetic disorder of lipoprotein metabolism. The lipidemic profile of the patients is characterized by very low levels of cholesterol and an almost complete absence of low-density (LDL), and VLDL lipoproteins [1,2,3,4,5]. The cause of the disease is the existence of a homozygous mutation in the gene encoding the 97-kDa subunit of the so-called Microsomal Triglyceride Transfer Protein (MTP). The cardinal function of MTP is to facilitate the transportation of phospholipids, triglycerides, and cholesterol esters between membranes, which is necessary for the formation of triglyceride droplets and the conversion of dense apoB-48 to mature VLDL. Therefore, the absence of MTP cancels the secretion of apoB lipoproteins, leading to fat and fat-soluble vitamin malabsorption [6,7,8]. MTP also transports lipids to antigen-presenting molecules on the surface of natural killer T cells, thus acting as a transmitter in several autoimmune conditions. MTP may, therefore, be considered a pharmacological target, the inhibition of which may achieve a reduction in plasma lipid levels and anti-inflammatory effects [9]. So far, more than 70 mutations have been described [10,11,12]. The heterozygote parents of patients with ABL exhibit normal lipid profiles.

The worldwide frequency of ABL has been estimated to be less than one case per 1 million individuals, with equal distribution in both sexes. It is of interest that a large proportion of the described cases are the progeny of consanguineous marriages [1], as it happened in our patients. The endoscopic picture of the upper gastrointestinal tract is not characteristic, while the histological examination of duodenal mucosa usually shows the presence of an abundance of lipid droplets [13,14]. The main clinical manifestations include symptoms of malabsorption, neurological manifestations, peripheral acanthocytosis, and visual disturbances [15], which are directly related to the severe malabsorption of lipids and lipid-soluble vitamins, especially vitamin E [16]. Patients with ABL also experience bone problems due to vitamin D deficiency and coagulopathy, which can lead to prolonged INR levels resulting from vitamin K deficiency [17].

To date, nearly 100 isolated cases have been published, and descriptions of long-term clinical outcomes are scattered [18]. This presentation aimed to describe the natural course and outcome of a unique Greek family with ABL who were followed up prospectively for 33 years.

## 2. Report of Cases

### 2.1. Long-Term Laboratory Follow-Up

#### 2.1.1. Lipidemic Profile

Table 1 shows the values of serum lipids at diagnosis and 33 years thereafter. As it is shown in the table, the lipid values remain unchanged. It should be stressed, however, that the absence of HDL was not complete. A significant observation was how low apoB levels were when measured in 2020 compared to those in 1990. They were almost 10–20 times lower compared to those seen in 1990 in all patients and despite the existence of this change Lp(a) levels did not change. Unfortunately, we cannot provide a reasonable explanation regarding the pathophysiology of this finding. We have also not been able to find similar data in the literature available to us.

#### 2.1.2. Liver Function Tests

The results of the liver function tests were normal in all patients. Only one patient (the female patient) showed a slight increase in serum transaminase values and cholestatic enzymes, while all other test results remained within normal limits. This fact supports the assumption that, contrary to the currently dominant opinion, MCT oil is not harmful to the liver, even after prolonged consumption.

#### 2.1.3. Serum Vitamin Levels

As expected, the levels of water-soluble serum vitamins were normal over time. The levels of serum inflammation markers (CRP, fibrinogen) were also normal. A somewhat unexpected finding was the increasing values of red cell MCV observed over time in all measurements despite normal levels of vitamin B12 and folic acid.

#### 2.1.4. Linkage Analysis

Linkage analysis is a statistical method used to map genes associated with inherited traits to their chromosomal locations. Genome-wide markers are tested in pedigrees segregating a trait. Linkage analysis combines this data to identify regions on the chromosomes that are likely to contain genes associated with this trait. Parametric linkage analysis is applied to cases of traits in which there is a Mendelian form of inheritance. The LOD score is used to identify gene positions.

**Figure 1:**
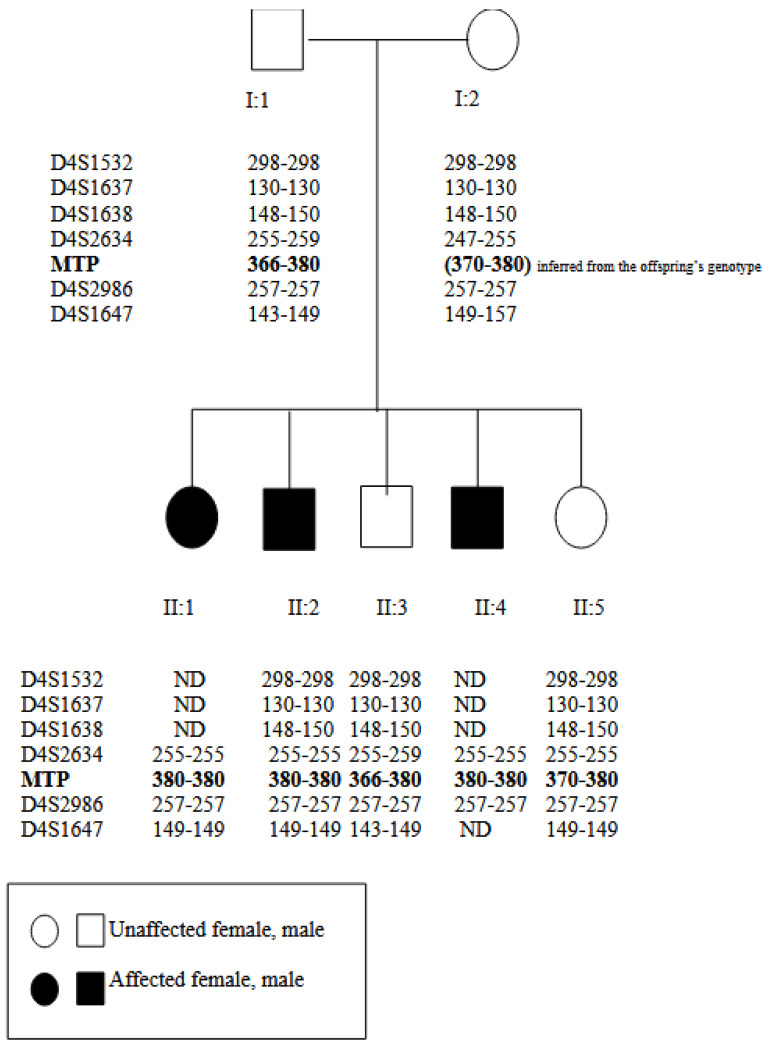
Results of linkage analysis (see text).

At the MTP locus, the two unaffected children (II:3 and II:5) carry a different allele to that of their affected siblings (II:1, II:2 and II:4). At loci D4S2634 and D4S1647, proximally and distally, respectively, the unaffected female (II:5) has a genotype similar to that of her affected siblings (II:1, II:2 and II:4) and different to that of her unaffected brother (II:3) (Figure 1).

#### 2.1.5. Mutation Analysis

Mutation analysis revealed that all three patients carry the P777X mutations in the homozygous state. However, for one of the unaffected offspring (II:5 in Figure 1), the linkage results suggest a disease phenotype and the mutation analysis revealed a heterozygous state. Combining both the linkage analysis and the mutation analysis data, we can assume that a double recombination event has occurred during meiosis.

#### 2.1.6. Cytogenetic Analysis

**Figure 2:**
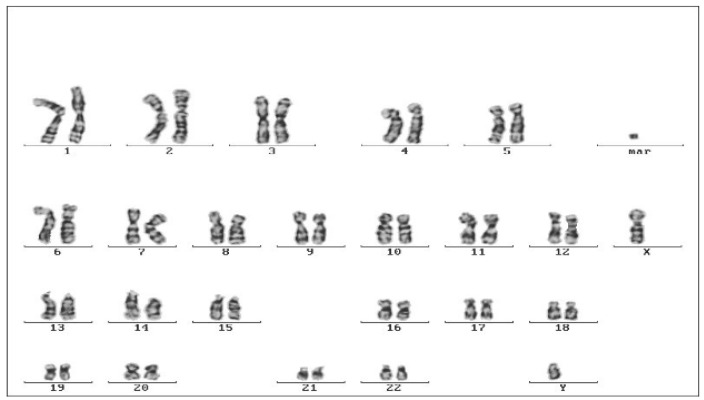
Karyotype in patient 1.

Cytogenetic analysis revealed normal karyotypes in all patients (Figure 2). Neither clonal nor sporadic chromosome abnormalities were detected. Only in the case of one patient (brother), an aberrant metaphase was detected. This cell was bearing a small, fragment-like acentric chromosomal material that seemed to be an extrachromosomal structure rather than a fragment belonging to a deleted chromosome. The abnormality cannot be characterized as clonal in the present study, since it was detected in only one cell. However, its presence in the karyotype could be of importance in the clinical/pathological picture, especially if it could be detected as a clonal aberration found in at least two cells.

#### 2.1.7. Fibrinogen Marker

**Figure 3:**
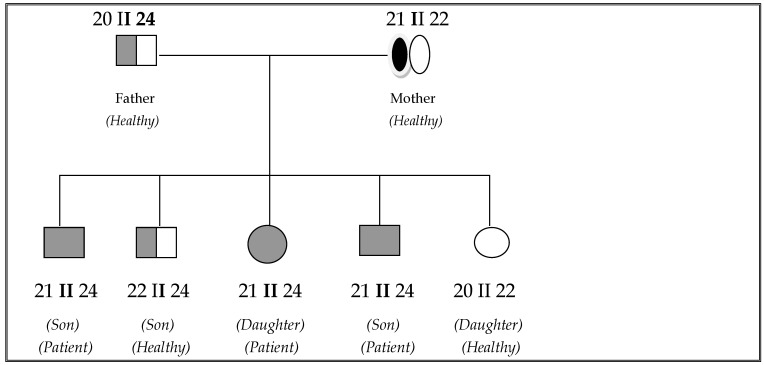
Fibrinogen-alpha at 4q28 marker in the healthy and diseased members of the family.

The authors analyzed chromosome 4, including the MTP gene (4q22-24), using short tandem repeat markers. One of the chromosome 4 markers tested, FGA (fibrinogen alpha at 4q28), shows complete correlation among all three patients, who inherited the same maternal and paternal FGA alleles. In contrast, the healthy siblings exhibit different allelic constellations (Figure 3). The proband inherited only maternal alleles on chromosome 4q, spanning a 150-centimogran region, consistent with segmental maternal isodisomy of 4q21-35 due to mitotic recombination. This maternal isodisomy (maternal UPD 4q) explains the homozygosity of the MTP gene mutation observed in the proband. The full correlation indicates that ABL and MTP (at 4q22-24) represent the same generic locus, eliminating the need for further distinction between them. Based on these findings, we can conclude that the family in our study represents the classical form of ABL, which lends particular interest to the observed association with HLA–B 18. The Gc and MNSs genes were not informative in this analysis.

#### 2.1.8. HLA Pattern

The HLA pattern revealed that the two patients with the most severe disease were homozygous for HLA antigen B18, whereas the third patient with milder symptoms had no alleles for B18.

#### 2.1.9. Anthropometric Parameters

Table 2 presents the main anthropometric parameters at the time of diagnosis and 33 years thereafter. As shown in the table, the majority of these parameters did not show striking alterations, except for the total skin fold thickness, which showed an 11% to 15% increase compared to the initial estimate.

#### 2.1.10. Current Clinical Situation

Table 3 summarizes the current clinical situation of the three patients. As indicated in the table, all patients experienced one to three bowel movements per day, with no other digestive system complaints. However, they suffered from quite prominent neurological and visual symptoms, including ataxia, paraplegia, and severe gait disturbances. Moreover, their vision status was heavily deteriorated, especially in the third patient, who had near-complete loss of vision in the right eye. Concerning drug consumption, the second patient was regularly receiving β-blockers and diuretics, while the third patient had been receiving anti-epileptic drugs in the last three years. Two key lessons from this remarkable family are that patient follow-up should be as close and systematic as possible, and diagnosis should occur before lesions become permanent. The unfavorable element in the described family is that they lived in a small provincial town in Greece where access to specialized medical services is limited.

The types and doses of vitamins received administered to the patients over the years are shown in Table 4.

**Figure 4:**
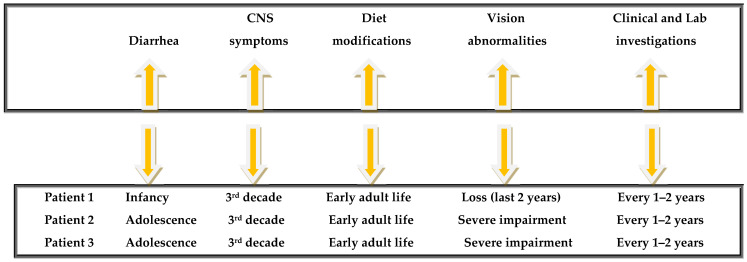
Timeline graph summarizing key disease milestones.

Finally, a time-line graph summarizing the most important clinical milestones is shown in Figure 4.

## 3. Discussion

ABL is an inherited disorder of β-apolipoprotein creation and secretion, transmitted by a single autosomal recessive gene [19]. It is a consequence of the appearance of mutations in the gene encoding the large subunit of MTP [6,7,8,20,21]. The diagnosis is based on a genetic test (detection of a pathogenic mutation in the MTP gene), the plasma lipid profile, the neurological and ophthalmological findings, and the presence of acanthocytes in the peripheral blood. So far, more than 74 MTP mutations have been identified in ABL patients [1]. Yang et al. found that ABL was inherited as a homozygous intron nine splice acceptor G(-1)-to-A mutation of the transfer protein gene. Maternal isodisomy was the basis for homozygosity of the MTP gene mutation [22]. Wang and Hegele studied six Canadian subjects with ABL and found that four of them were single homozygotes and two heterozygotes for MTP gene mutations [23]. Ohashi et al. [9] have screened mutations of the MTP gene in four patients with ABL. They identified three novel mutations: a frameshift mutation caused by an adenine deletion at position 1389 of the cDNA, a missense mutation, Asn780Tyr (homozygous form), and a splice site mutation, 2218-2A-->G (heterozygous form) mutations that predicted to encode truncated forms of MTP. Walsh et al. [8] identified a novel missense mutation (D169V) in a 4-month-old Turkish male child with ABL. They concluded that this mutation is detrimental, as it disrupts an internal salt bridge, resulting in the loss of protein disulfide isomerase binding and lipid transfer. Khatun et al. [7] found that the mutations R540H and N780Y lacked phospholipid transfer activity along with the already known deficiency in triglyceride transfer activity. Novel mutants S590I and G746E also did not transfer triglycerides and phospholipids in contrast with D384A mutations that displayed both triglyceride and phospholipid transfer activities. Zamel et al. [4] detected a homozygous frameshift mutation in exon 13 of MTP due to a single nucleotide base-pair deletion in one of their two patients. The second one was a compound heterozygote for known pathogenic mutations (c.2237G>A (p.G746E) and c.2524A>T (p.K842X). ABL should be distinguished from another family of genetic disorders, namely Familial Hypobetalipoproteinemia (FHBL1, OMIM 615558), in which both homozygotes and heterozygotes have symptoms identical to ABL and hypobetalipoproteinemia with selective depletion of B48 apolipoprotein (chylomicron retention disease). In contrast with FHBL, obligate heterozygote parents of patients with ABL have normal plasma lipoprotein profiles. Two out of three patients showed a homozygous pattern for HLA B18, while the third patient, in whom the disease was running with milder symptoms, had no alleles for B18. No association between any particular HLA type and this disorder could be demonstrated.

Regarding the histological characteristics of ABL (e.g., enterocyte vacuolization), it should be emphasized that they are not specific to the disease, as they can also be observed in other conditions, such as megaloblastic anemia, celiac disease, and tropical sprue [24], although different views exist considering the presence of acanthocytes as a key element for the correct diagnosis of the disease [25].

Dietary modifications were implemented in our patients to reduce steatorrhea and alleviate neurological and ocular manifestations. The nutritional guidelines recommended limiting dietary fat intake and supplementing it with MCT oil, as well as increasing protein and high-calorie food consumption. Oral essential fatty acid supplementation, such as one teaspoon of olive oil rich in polyunsaturated fatty acids per day, was applied. MCTs, due to their unique characteristics of digestion, absorption, and oxidation, play a significant role in the management of several gastrointestinal disorders, either alone or as part of an enteral product. The need for bile or pancreatic enzymes in the gut for the absorption of MCTs makes them a crucial source of calories in cases of malabsorption and steatorrhea. MCTs are rapidly metabolized, as they do not require the carnitine acyltransferase system for transport into the mitochondria for β-oxidation.

Patients with ABL develop progressive pigmentary retinopathy due to either increased peroxidation of unsaturated fatty acids contained in phospholipids of myelin (due to vitamin E deficiency) or to secondary alterations in the structure and synthesis of the retina (because of the low levels of plasma lipids) [26]. In our patients, vision disturbances got worse over time, leading to almost complete blindness in one of the patients and severe impairment in the other.

During the follow-up, most of the hematological parameters, including hematocrit, hemoglobin, fibrinogen, ferritin, vitamin B12, Vitamin 25(OH)2D3, and folic acid, remained stable and close to the normal limits. However, no improvements in the levels of HDL, LDL, or triglycerides were observed.

Concerning the lipid-soluble vitamin K, it is well established that all malabsorption syndromes, including ABL, may cause vitamin K deficiency because the solubilization of fat must precede the absorption of vitamin K. In vitamin K-deficient states, the levels of vitamin K-dependent plasma proteins are nearly normal, although their functions in reactions that require a phospholipid surface are impaired. A regular diet provides 300–500 μg of vitamin K, a significantly larger amount than the standard requirement of 1 μg/d. Vitamin K supplementation in this disorder requires high doses, which was applied in our patients. Although the levels of vitamin K in patients with ABL were low due to fat malabsorption and, consequently, the levels of some vitamin K-dependent plasma proteins were below the normal limits, no clinically significant consequences related to blood coagulation have been observed, even after many years of follow-up. However, we suggest that in patients with ABL, parenteral administration of vitamin K is indicated to correct PT and to improve the levels of some vitamin K-dependent coagulation factors.

On the other hand, vitamin E should be administered for an extended period in very high doses (2400 to 12,000 IU per day), as in these doses, it can be absorbed via portal circulation. Early therapy with vitamin E can improve neurological dysfunction and stabilize neurological status. It is of interest that arterial atherosclerotic lesions are rarely found in patients with ABL [3]. Despite some theoretical arguments, most agree that serum vitamin E levels can be used to monitor compliance and adequacy of therapy [17,27]. Vitamin E absorption can exacerbate the deficit in vitamin K.

Vitamin D represents an essential lipid-soluble vitamin for patients with ABL. Administration of 1000 mg/d should be considered in all ABL patients. The low levels of vitamin A could be normalized if it is administered in high doses (100–400 IU/Kg/d).

In general, the clinical symptoms that regressed or improved in the three patients were primarily related to nutritional deficiencies rather than those affecting the retinal or central nervous systems. It is interesting that in our female patient, the manifestations from the reproductive system were minimal, as despite the absence of LDL, the levels of HDL could be sufficient for the synthesis of steroid hormones, including sex hormones. Despite the relatively low levels of progesterone, the patient became pregnant. After extensive discussion with the patient, an abortion was carried out in the third month. It is now accepted that reduced fertility in women represents an unwanted complication of the disease. If gestation happens, then sufficient vitamin supplementation might be a significant problem, along with the possible teratogenic effects of the disease on the fetus and the possible maternal complications [28].

Finally, disturbances of liver function in patients with ABL have been previously described as a consequence of hepatic steatosis [29]. Fatty liver, cirrhosis, and hepatocellular carcinoma have been reported in familial hypobetalipoproteinemia and ABL, probably due to decreased triglyceride export from the liver. Moreover, early reports also suggested that MCT oil use in ABL was associated with hepatic fibrosis and steatosis, ultimately leading to the development of micronodular cirrhosis [30]. For this reason, some textbooks have recommended avoiding MCT oil in ABL, and many clinicians still hold this belief. However, the long period of experience with MCT oil seen in this group of patients without serious hepatic complications is interesting and informative, suggesting that fibrosis observed in other cases may have been related to the general evolution of the disease and probably unrelated to MCT oil. The liver ultrasound performed on the female patient showed a bright liver compatible with lipid accumulation. Serum transaminases and lactic dehydrogenase were slightly above normal limits in the female patient, while they were normal in the other two.

In our series of patients, the second patient was taking antihypertensive medications and a blood thinner because of an underlying cardiovascular disorder, which is very unusual in ABL. The third patient was also taking valproic acid due to seizure disorder, possibly as a result of neurological involvement.

## 4. Conclusions

In conclusion, ABL is a very rare disorder with significant consequences for many organs and systems. Mainstays of treatment include strict adherence to a low-fat diet, supplementation with essential fatty acids, and high oral doses of fat-soluble vitamins. The adoption of a special diet, including MCT oil, improves both the anthropometric parameters and the symptoms of malabsorption. A modified diet could also prolong survival for at least the first 6 decades of life. The prolonged survival of these patients is due to the adoption of dietary restrictions, including the continuous administration of MCT oil and close surveillance. One of the main conclusions of this description is the documentation of the long-term safety of medium-chain triglyceride intake. Long-term administration of MCT oil does not appear to pose a risk to liver function. However, clinicians should be cautious regarding the last conclusion, as this was drawn from observations in only three patients from the same family.

## Figures and Tables

**Table 1:** Serum lipidemic profile at diagnosis and 33 years thereafter (the values in parentheses correspond to the normal range).

Lipidemic Profile/ Year	Patient 1		Patient 2		Patient 3	
	1990	2023	1990	2023	1990	2023
Cholesterol (<200 mg/dL)	46	57	65	40	43	39
Triglycerides (<150 mg/dL)	0	0	0	0	0	0
High Density Lipoproteins (>60 mg/dL)	43	58	61	40	41	46
Low Density Lipoproteins (<100 mg/dL)	0	0	0	0	0	0
Apo A-1 Lipoprotein (119–228 mg/dL)	63	70.2	76	55.3	56	48
Apo B Lipoprotein (51–165 mg/dL)	20	1.2	20	2.2	20	3.1
Lp(a) Lipoprotein (<75 nmol/L)	1.3	1.7	0.8	1.8	1.6	1.9

**Table 2:** Over-time changes in certain anthropometric parameters of the three patients with ABL.

Anthropometric Parameter/Year	Patient 1 1990	Patient 1 2023	Patient 2 1990	Patient 2 2023	Patient 3 1990	Patient 3 2023
Body weight (Kg)	33.1	35.3	47.3	48.1	46.7	47.2
Height (cm)	154	153	170	168	165	165
Body mass index (BMI)	13.4	15.2	15.5	16.7	15.8	16.9
Body fat content (%)	21.0	30.0	14.0	16.0	13.0	13.0
Mid arm circumference (cm)	19.5	20	21	21	22.5	22.5
Skin fold thickness (SFT) in back of upper arm (triceps) (mm)	6	7	4	5	3	3.5
SFT in front of upper arm (biceps) (mm)	4	5	2	2.5	2	3
SFT in subscapular (mm)	5	5	8	8	5	5.5
SFT in suprailiac (waist) (mm)	6	6	3	4	3	3
Total SFT (mm)	21	23 (10.9%)	17	19.5 (14.7%)	13	15 (15.4%)

**Table 3:** Current (2023) patients’ clinical situation and drug therapy.

Clinical Parameter	Patient 1	Patient 2	Patient 3
Bowel movements/ abdominal symptoms	Three per day normal or semi formed, absence of abdominal symptoms.	One to three normal abdominal pain, increased bowel sounds.	One to two per day normal, absence of abdominal symptoms.
Neurological manifestations	Clinical deterioration, ataxia.	Paraplegia, gait with mechanical help.	Clinical deterioration.
Nutritional support	MCT Oil Module 500 mL; various dietetic supplementations, including “Abound 24g”: (powder containing mainly aminoacids such as arginine and glutamine), “Fortimel extra” (containing carbohydrates, aminoacids, and 19% fat), and “Fresubin”. These products were received continuously throughout the follow-up period.	MCT Oil Module 500 mL; various dietetic supplementations including “Abound 24g”: (powder containing mainly aminoacids such as arginine and glutamine), “Fortimel extra” (containing carbohydrates, aminoacids, and 19% fat), and “Fresubin”. These products were received continuously throughout the follow-up period.	MCT Oil Module 500 mL various dietetic supplementations including “Abound 24g”: (powder containing mainly aminoacids such as arginine and glutamine), “Fortimel extra” (containing carbohydrates, aminoacids and 19% fat), and “Fresubin”. These products were received continuously throughout the follow-up period.
Vision status	Vision impairment drowsiness, inability to read.	Significant vision impairment.	Almost complete right blindness with only light perception; macular degeneration.
Pharmaceutical treatment	Ferrum per os, Vit D.	Metoprolol, Apixaban, Eplerenone, Furosemide, Levothyroxin.	Depakin (valproic acid).

**Table 4:** Vitamin supplementation regimens (*) and dosages during the follow-up.

Vitamins	Patient 1	Patient 2	Patient 3
A	100 IU/KgBW/d corresponding to 5000 IU/d (**)	100 IU/KgBW/d corresponding to 5000 IU/d	100 IU/KgBW/d corresponding to 5000 IU/d 100
D	800–1200 IU/d (1 tab 1000 IU/d)	800–1200 IU/d (1 tab 1000 IU/d)	800–1200 IU/d (1 tab 1000 IU/d)
E	100 IU/KgBW/d (at least 5000 IU/d)	100 IU/KgBW/d (at least 5000 IU/d)	100 IU/KgBW/d (at least 5000 IU/d)
K	5 mg 2 times per week	5 mg once a week	5 mg once a week
Iron, folate, Vit B_12_	Iron and vit b12 and folic acid were supplemented only in this patient, as they had the most severe symptoms.	In the large majority of examinations, the levels were normal.	In the large majority of examinations, the levels were normal.

(*) In Greece, a variety of preparations are available on the market. These preparations must be purchased by the patients themselves because the NHS does not cover their purchase. Therefore, the preparation may change periodically due to the patients’ circumstances. (**) The lowest dose was chosen to avoid side effects from overdose.

## Data Availability

Data concerning the results presented in this scientific paper are available to readers after an official letter to te corresonding author, J. K. Triantafillidis.
